# Supplementary figures and images for: A machine learning model trained on a high-throughput antibacterial screen increases the hit rate of drug discovery
Source: PLoS Comput Biol. 2022 Oct 13;18(10):e1010613. doi: 10.1371/journal.pcbi.1010613 (PMC9624395; doi:10.1371/journal.pcbi.1010613)

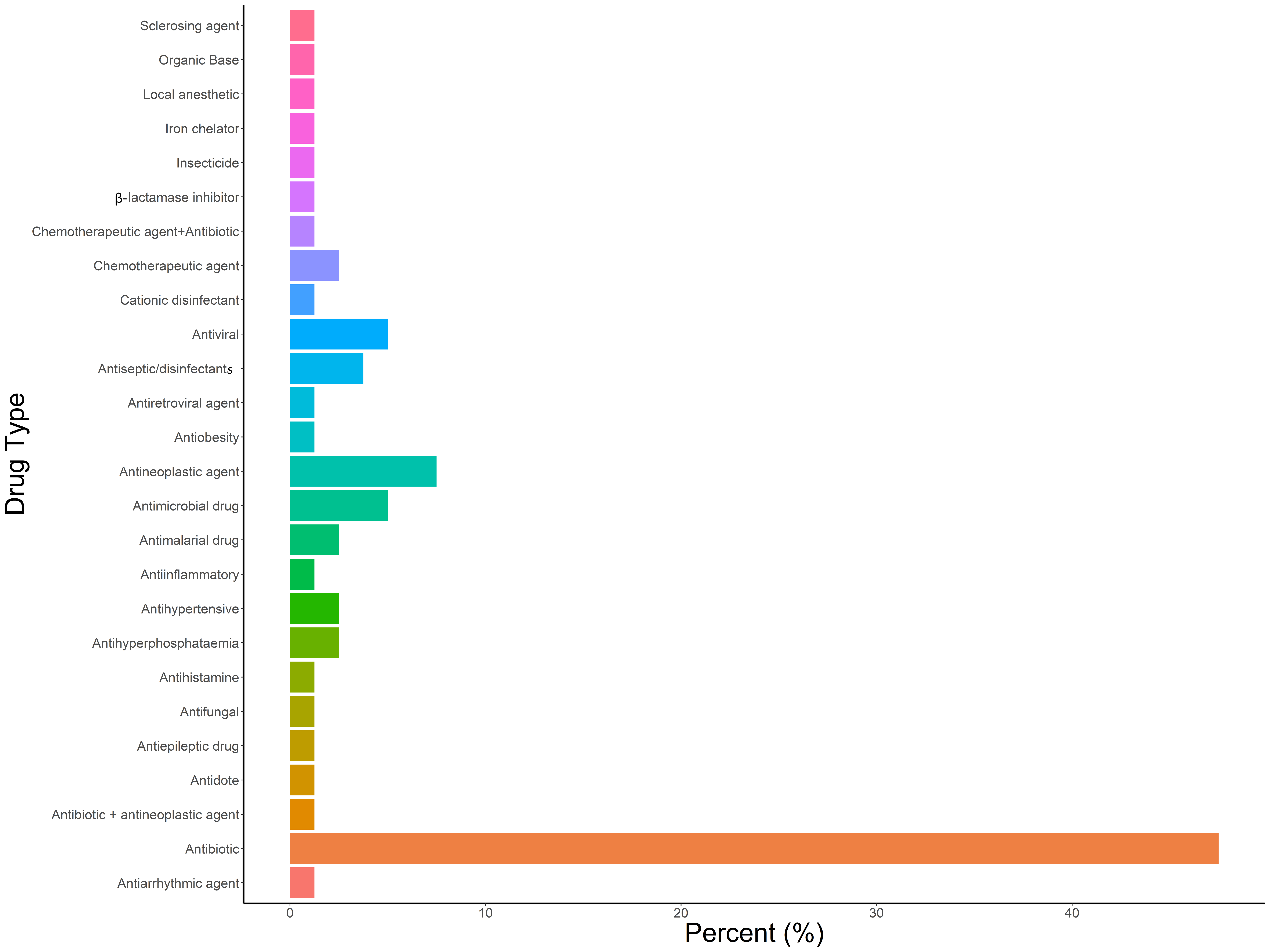

Supplement: S1 Fig — (TIF) [file pcbi.1010613.s001.tif]

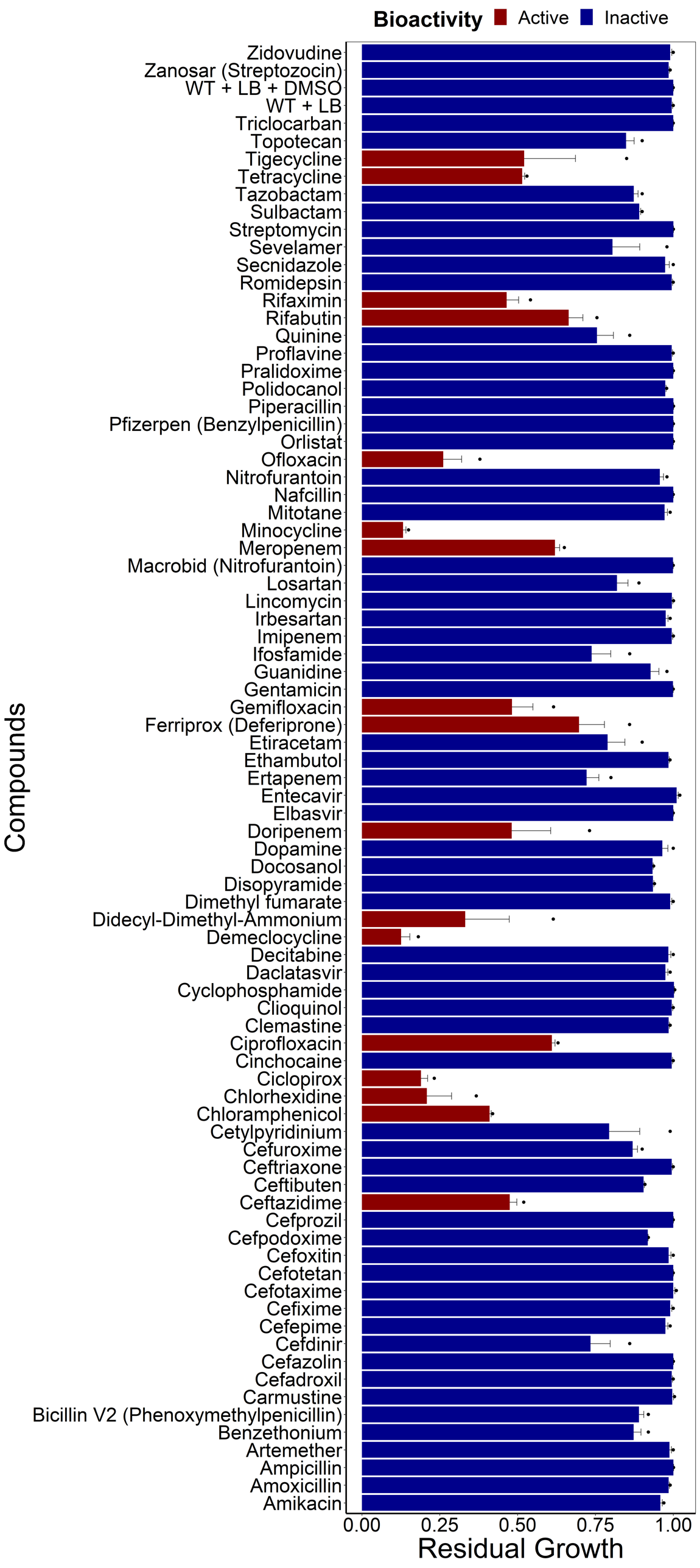

Supplement: S2 Fig — The screening identified 17 bioactive compounds with a positive predictive value (PPV) of 25.9%. The activity of growth inhibitory and non-growth inhibitory compounds are shown in red and blue, respectively. Results are the average of three independent biological replicates. Error bars indicate mean ± SD. (TIFF) [file pcbi.1010613.s002.tiff]

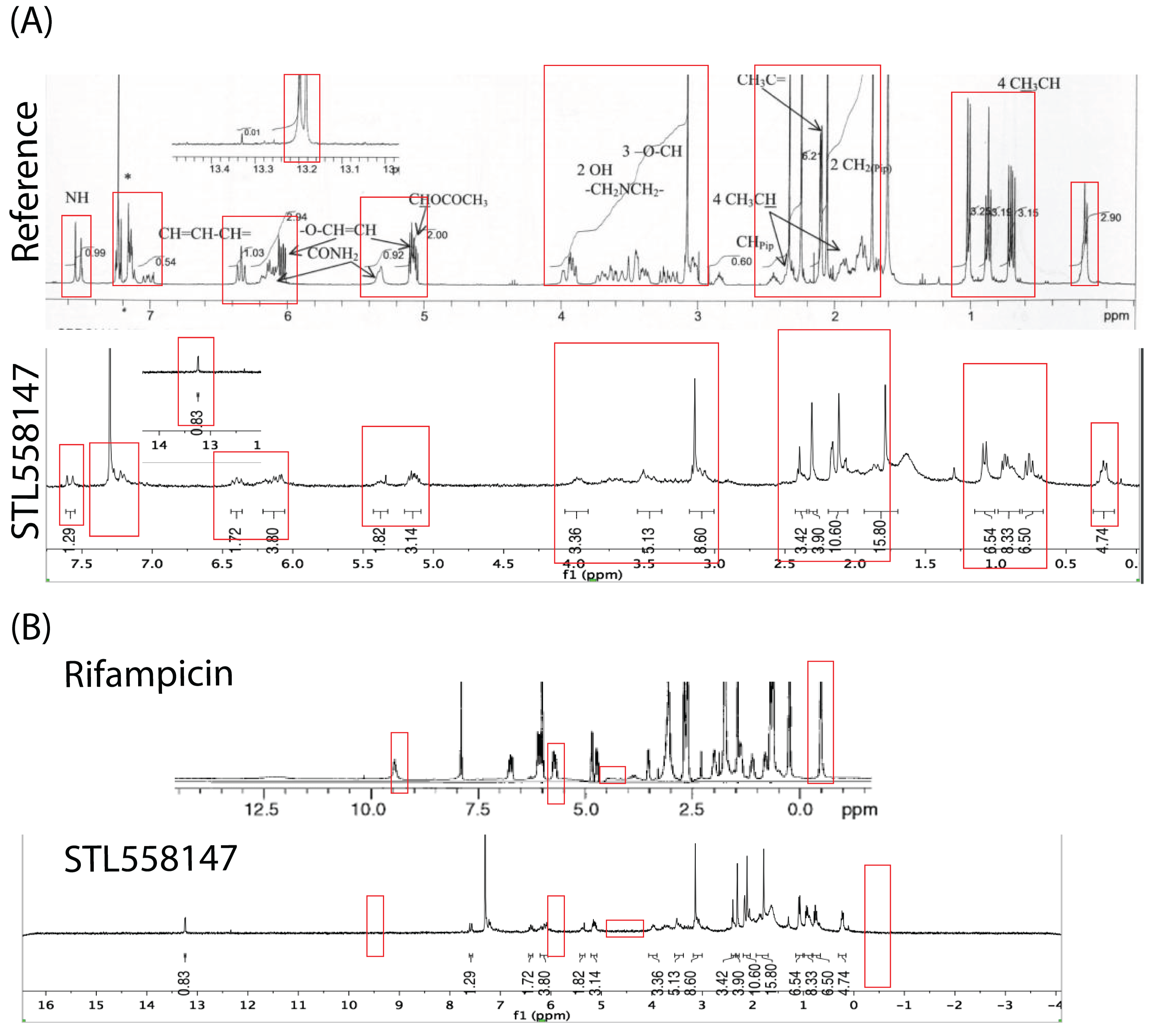

Supplement: S3 Fig — (A) Comparison of STL558147 NMR spectrum with the reference spectrum provided by the supplier. The peaks in the reference spectrum are all accounted for in our NMR (highlighted by red boxes). (B) Comparison of STL558147 and rifampicin NMR spectra [57]. The differences are highlighted with red boxes. (TIF) [file pcbi.1010613.s003.tif]

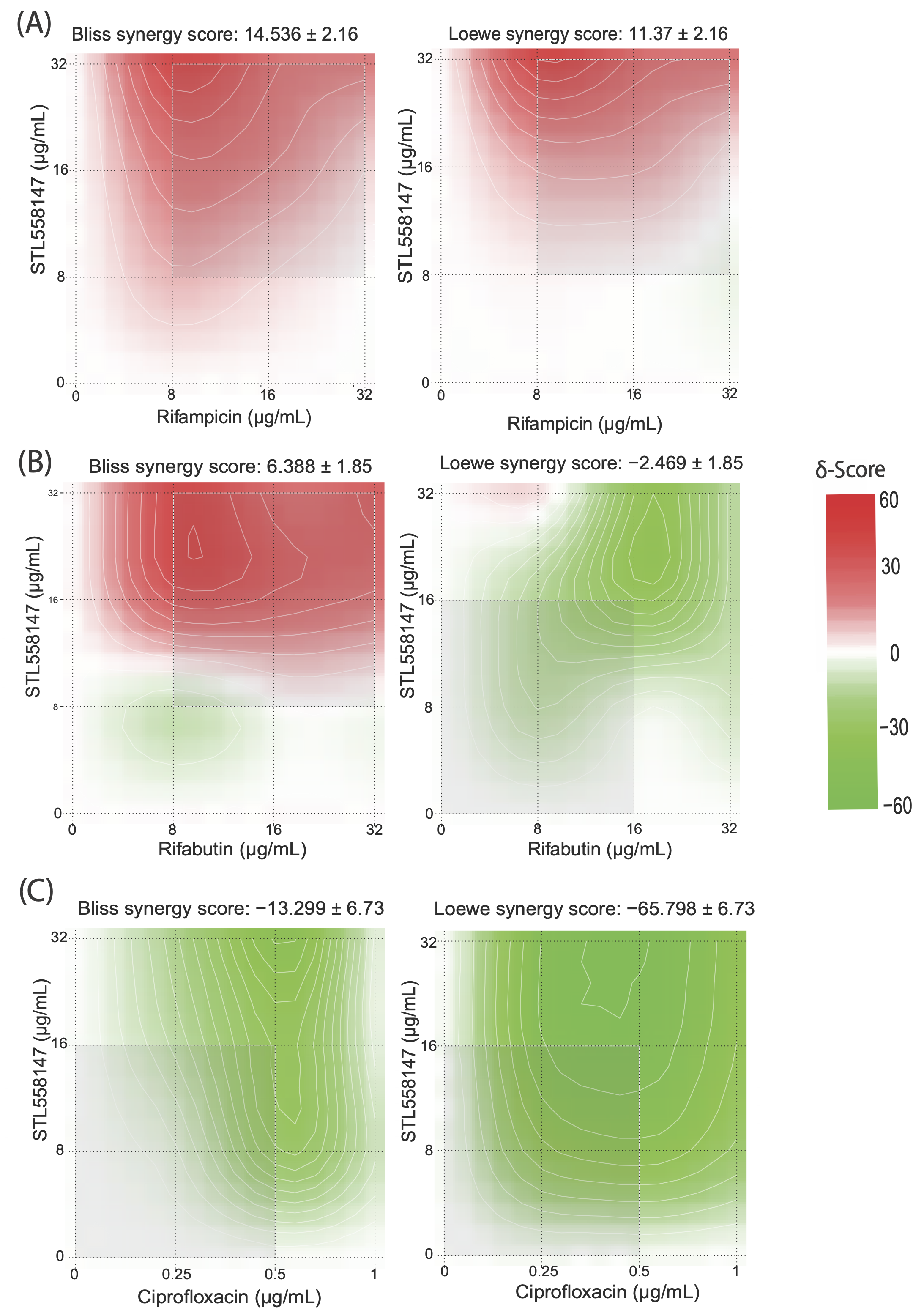

Supplement: S5 Fig — STL558147 displayed additive interactions with rifampicin (A) and rifabutin (B), whereas exhibited antagonistic interaction with ciprofloxacin (C). The most synergistic area in each combination is highlighted with a rectangular box inside the plot. Green (negative δ-scores) indicate antagonistic interactions, and red (positive δ-scores) indicate synergistic interactions. Synergy scores >15, between -5 to 15, and < -15 were considered synergistic, additive, or antagonistic, respectively. Results are the average of at least three independent biological replicates. Synergy scores are shown as mean ± SEM. Synergy scores were calculated using SynergyFinder 2.0 [31]. (TIFF) [file pcbi.1010613.s005.tiff]

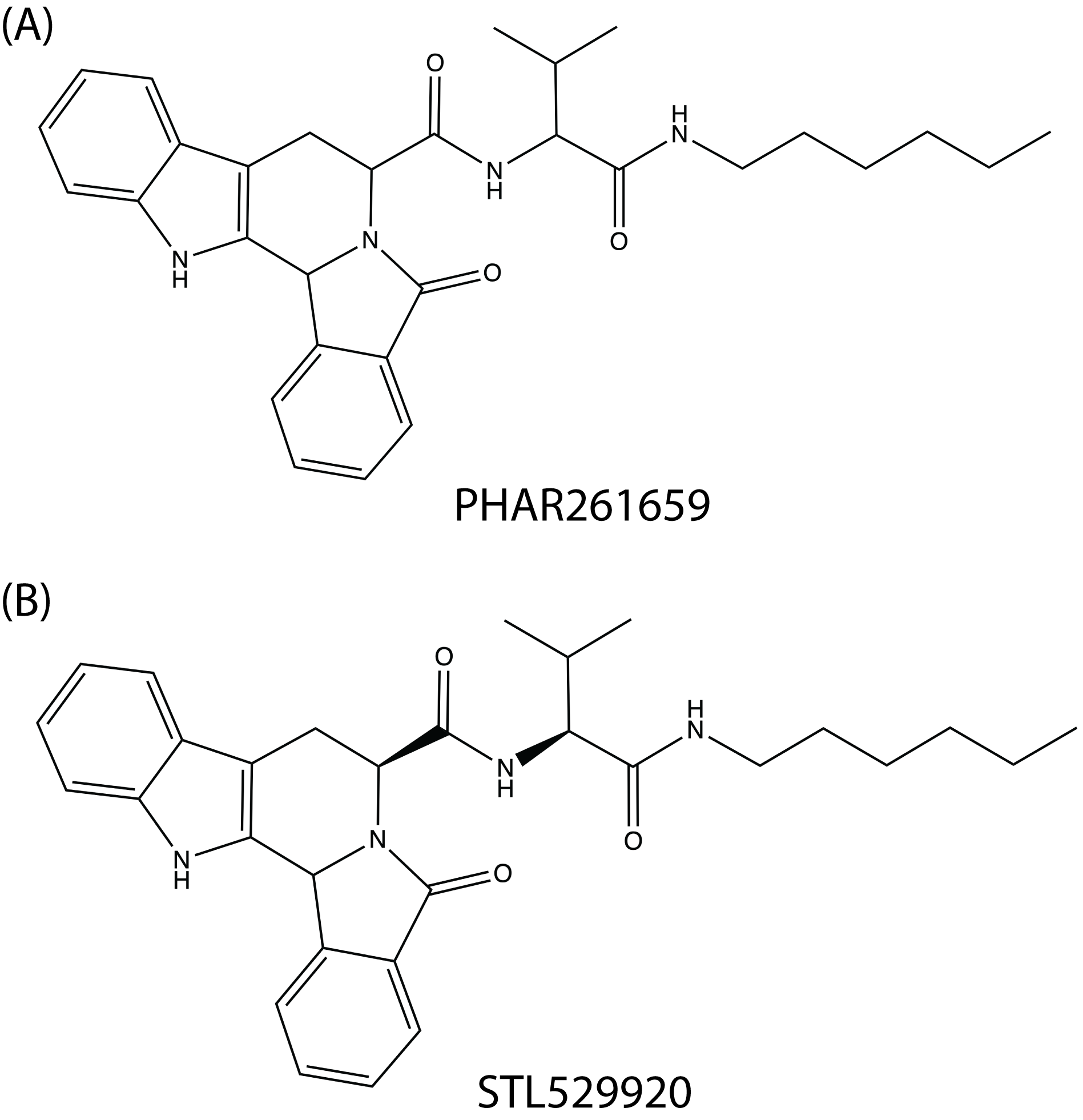

Supplement: S6 Fig — Structures of PHAR261659 (A) and STL529920 (B). STL529920 is a stereoisomer of PHAR261659. (TIF) [file pcbi.1010613.s006.tif]

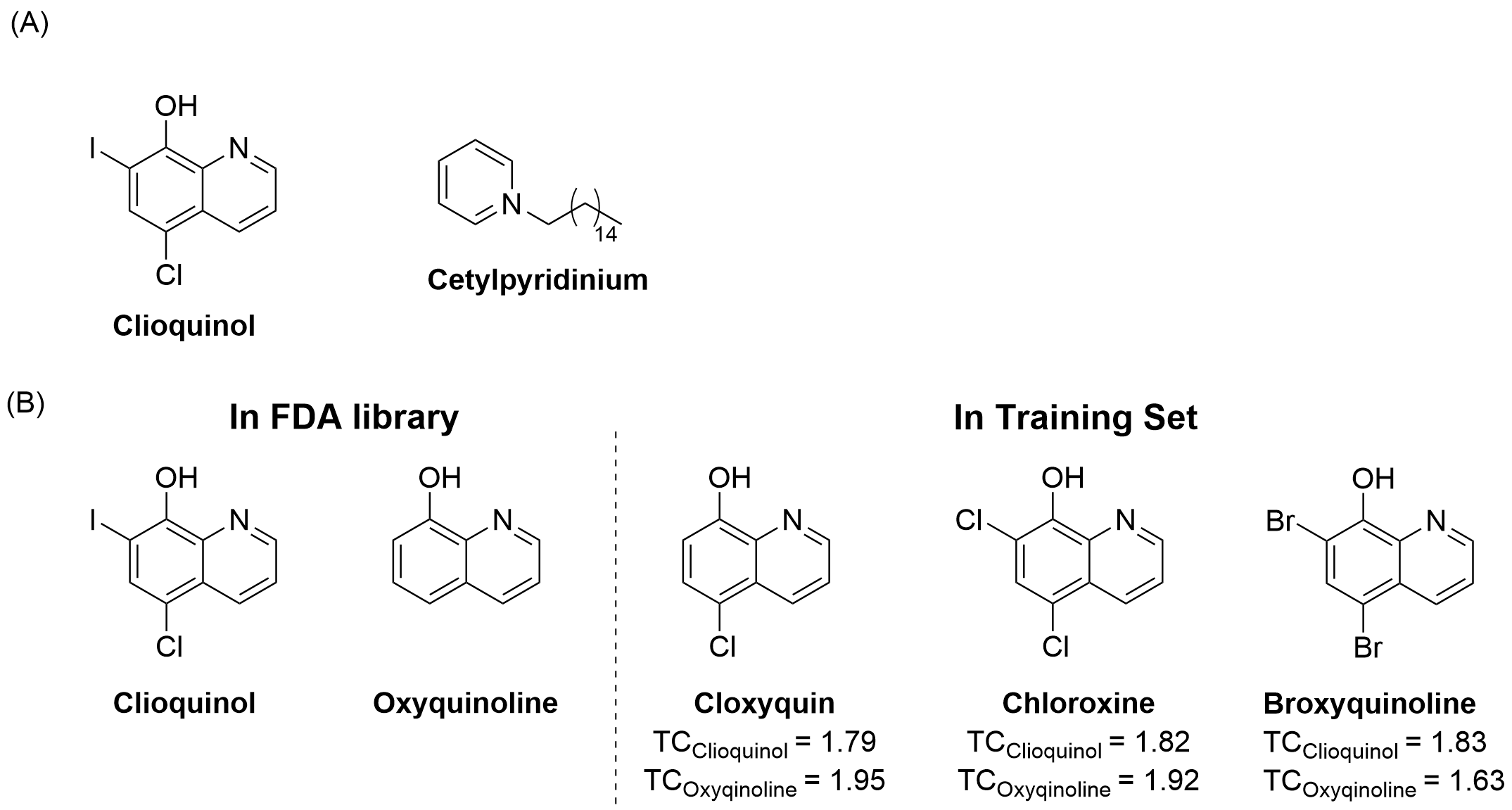

Supplement: S7 Fig — The score is a range between 0 and 2 with 2 being full color and shape overlap and 0 being no overlap. (a) Two molecules (Clioquinol and Cetylpyridinium) in the FDA-approved library were also present in the HTS dataset set (i.e., TC score of 2). (b) Two molecules in the FDA-approved library (Oxyquinoline and Clioquinol) had a similar structure to three molecules in the HTS dataset (Chloroxine, Cloxyquin and Broxyquinoline). (TIF) [file pcbi.1010613.s007.tif]
