# Supplementary material for: A machine learning model trained on a high-throughput antibacterial screen increases the hit rate of drug discovery
Source: PLoS Comput Biol. 2022 Oct 13;18(10):e1010613. doi: 10.1371/journal.pcbi.1010613 (PMC9624395; doi:10.1371/journal.pcbi.1010613)
Supplement: S9 Table — (PDF) [file pcbi.1010613.s016.pdf]

Table S9: Synergy score for STL558147 in combination with other clinically used antibiotics.

Drug Combination	Synergy Score			
	**Bliss**	**Loewe**	**ZIP**	**HSA**
Tobramycin - STL558147	2.29	-11.33	2.36	-4.65
Ciprofloxacin - STL558147	-13.30	-65.80	-13.68	-16.12
Chloramphenicol - STL558147	0.94	-15.11	3.80	3.52
Meropenem - STL558147	-0.44	-1.84	1.38	-2.06
Ceftazidime - STL558147	40.99	38.94	41.04	38.94
Novobiocin - STL558147	1.21	2.91	1.26	2.91
Colistin - STL558147	52.07	39.53	51.55	39.71
Polymyxin B - STL558147	37.97	24.19	38.32	24.70
Chlorhexidine - STL558147	5.21	2.28	5.32	2.92
Rifampicin - STL558147	14.54	11.37	14.77	11.69
Gemifloxacin - STL558147	-0.48	-0.96	-0.69	-3.94
Rifabutin - STL558147	6.39	-2.47	6.38	12.41
Ceftazidime – Avibactam (Control)	39.90	33.72	41.20	33.80
