# Supplementary material for: A machine learning model trained on a high-throughput antibacterial screen increases the hit rate of drug discovery
Source: PLoS Comput Biol. 2022 Oct 13;18(10):e1010613. doi: 10.1371/journal.pcbi.1010613 (PMC9624395; doi:10.1371/journal.pcbi.1010613)
Supplement: S10 Table — (PDF) [file pcbi.1010613.s017.pdf]

Table S10: Analogs of PHAR261659 (related to Figure 3B and Figure 5)

ZINC ID	MolPort ID	Compound ID	logP	Predicted Score
ZINC000012887434	MolPort-002-533-167	STL530142	2.541	0.318008609
ZINC000038139907	MolPort-002-532-082	PHAR360228	2.854	0.287549686
ZINC000012887666	MolPort-002-533-261	STL532397	2.931	0.315283336
ZINC000012887826	MolPort-002-533-311	STL530691	3.074	0.242500985
ZINC000008791565	MolPort-002-533-177	STL533461	3.321	0.209535618
ZINC000012887180	MolPort-002-533-016	STL530024	3.464	0.184009221
ZINC000012887391	MolPort-002-533-128	STL530088	3.71	0.367519242
ZINC000008791680	MolPort-002-533-366	STL530883	3.837	0.090382348
ZINC000012887837	MolPort-002-533-321	STL533319	3.839	0.287990698
ZINC000008791658	MolPort-002-533-324	STL531120	3.941	0.158984759
ZINC000008791692	MolPort-002-533-388	STL529724	4.085	0.38078694
ZINC000070701508	MolPort-002-532-875	STL532790	4.1	0.270701405
ZINC000008791064	MolPort-002-532-342	STL532944	4.228	0.066577572
ZINC000012883759	MolPort-002-531-714	STL531811	4.618	0.053563598
ZINC000008876405	MolPort-038-428-235	PHAR261659	4.475	0.497985628
ZINC000008876407	MolPort-002-534-068	STL529920	4.475	0.486795527
