# Supplementary material for: A machine learning model trained on a high-throughput antibacterial screen increases the hit rate of drug discovery
Source: PLoS Comput Biol. 2022 Oct 13;18(10):e1010613. doi: 10.1371/journal.pcbi.1010613 (PMC9624395; doi:10.1371/journal.pcbi.1010613)
Supplement: S11 Table — (PDF) [file pcbi.1010613.s018.pdf]

S11 Table. Bacterial strains and plasmids used in this work.

Strains	Features	Source/Reference
Burkholderia cenocepacia K56-2	Clinical isolate from cystic fibrosis patient; ET12 lineage	[1]
Pseudomonas aeruginosa PAO1	Human wound isolate	[2]
Acinetobacter baumannii 1225	Clinical isolate, coccyx	[3]
Enterobacter cloacae ENT001_EB001	Wild-type	From Dr. Ayush Kumar, University of Manitoba
Methicillin-resistant *Staphylococcus aureus* (MRSA) subsp. *aureus* ATCC33592	Gentamicin and methicillin resistant, *pvl* negative	Received from the American Type Culture Collection (ATCC)
Klebsiella pneumoniae ESBL_120310	Extended-spectrum β-lactamase positive	From Dr. George G Zhanel, University of Manitoba [4]

References

1. Darling P, Chan M, Cox AD, Sokol PA. Siderophore production by cystic fibrosis isolates of *Burkholderia cepacia*. Infection and immunity. 1998;66: 874–877.

2. Holloway BW, Zhang C. Genetic maps. In: Locus maps of complex organisms. 5th ed. Cold Spring Harbor: Cold Spring Harbor Laboratory Press; 1990.

3. Weber BS, Miyata ST, Iwashkiw JA, Mortensen BL, Skaar EP, Pukatzki S, et al. Genomic and Functional Analysis of the Type VI Secretion System in *Acinetobacter*. Cascales E, editor. PLoS ONE. 2013;8: e55142. doi:10.1371/journal.pone.0055142

4. Denisuik AJ, Karlowsky JA, Adam HJ, Baxter MR, Lagacé-Wiens PRS, Mulvey MR, et al. Dramatic rise in the proportion of ESBL-producing *Escherichia coli* and *Klebsiella pneumoniae* among clinical isolates identified in Canadian hospital laboratories from 2007 to 2016. Journal of Antimicrobial Chemotherapy. 2019;74: iv64–iv71. doi:10.1093/jac/dkz289
