# Supplementary material for: A machine learning model trained on a high-throughput antibacterial screen increases the hit rate of drug discovery
Source: PLoS Comput Biol. 2022 Oct 13;18(10):e1010613. doi: 10.1371/journal.pcbi.1010613 (PMC9624395; doi:10.1371/journal.pcbi.1010613)
Supplement: Attachment — Submitted filename: Response to Reviewers Comments_V2.pdf [file pcbi.1010613.s019.pdf]

Response to Reviewers

Reviewer #1: This is an excellent paper and for once I have absolutely no comments. It is well written, nicely presented and will become a reference in the field. They also acknowledge previous publications appropriately.

We thank the reviewer for taking the time and effort to assess our manuscript.

Reviewer #2: This is an interesting study. The authors built a machine learning model with antibacterial dataset, which was further used for high-throughput screening. The hit rate proved to be increased. I appreciate using Bayesian optimization for hyperparameter optimization. The predictions were further evaluated with experiments. The manuscript is well-written and I would suggest to accept this manuscript after minor revision.

Here are some questions that I have.

1. On page 18, there are some evaluation metrices reported. Can you also add the results of Matthews correlation coefficient (MCC). The advantages of MCC is reported in https://bmcgenomics.biomedcentral.com/articles/10.1186/s12864-019-6413-7.

We thank the reviewer for this great suggestion. We have now added the Matthews correlation coefficient (MCC) to our binary classification results in addition to ROC-AUC, PRC-AUC, and F1 score. We have added the MCC analysis to Tables S1, and S2, and modified the related contents in the results section accordingly (see lines 118-119 in page 6). The definition of MCC was also added to the section "Evaluation Methods" in line 384-395 of page 16.

2. All the source codes were not listed in the manuscript, and it would be very helpful if they can be provided to support open science.

We thank the reviewer for the above suggestion. The source codes of the machine learning model (D-MPNN) used in our study were implemented by Yang et al. (https://doi.org/10.1021/acs.jcim.9b00237) and shared through the open-source package Chemprop (https://github.com/chemprop/chemprop). We thank the authors for sharing the code and providing detailed implementation and instructions in the repository. To further expand the

details of the 16 predictive models used in this study, we created our repository (https://github.com/cardonalab/Prediction-of-ATB-Activity) in which we included the trained weights, logs, library compounds similarity comparisons and results of our models. The link to the repository has also been added to the "Code and Data Availability" section at the end of the manuscript (see page 32, line 569-573)

Reviewer #3: In this manuscript, the authors trained an ML model with data from a high throughput screening experiment and used this ML model to predict compounds with antibacterial activity in the library of FDA-approved compounds and natural products. Then, some compounds with growth inhibitory activity against several Gram-negative bacteria were identified by wet experiments. This manuscript combines experiments and computation well and has the potential to be accepted. However, the reviewer has some concerns and suggestions.

Major Comments:

(1) The authors claimed many times in the article that their approach increases the hit rate of drug discovery by 12-fold at least. However, the hit rate of the virtual screen has a great relationship with the experimental system and the definition of hits. It is not appropriate to quantitatively compare the hit rate in this study with that of the hit rate from conventional whole-cell-based high-throughput screens. I recommend that the authors compare the hit rate in this study with the hit rate from their previously performed HTS (the training dataset).

We appreciate and agree with this thoughtful and important comment by the reviewer. The hit rate of the previously performed HTS was 0.67% (Selin et al., 2015). This hit rate was slightly modified after binarization by the B-score to 0.87%, The new hit rates obtained after prediction by the model and testing the top predicted compounds were 26% and 12% for the FDA-approved library and the natural product library, respectively. Therefore, our calculation of an increase in the hit rate with respect to the training set is 14-fold. We have modified this number and added an explanatory paragraph in the discussion (see page 11, lines 254-255 and 261-264).

(2) Most of the compounds screened by the ML model from the FDA-approved compound library are known antibiotics. Are there very similar molecules to those hit compounds in the training set?

Please provide the maximum similarity between these hit compounds and the compounds in the training set.

We appreciate this important point. The original HTS dataset contained known antibiotics, which are also present in the FDA-approved library. Before running the model to predict growth inhibitory activity in the FDA-approved library, we compared the HTS dataset with the FDA-approved library and compounds with identical SMILES strings were filtered out from the FDA-approved library. We clarified this aspect in page 6, lines 126-129. As per the reviewer's request we further investigated the maximum similarity between the two compound libraries, with the Openeye Scientific's Rapid Overlay of Chemical Structures (ROCS) program and calculated the Tanimoto Combo (TC) scores for each of the compounds present in the training set compared to each molecule present in the FDA library. This produced 332,484 TC scores. The most similar molecules (TC > 1.8) were then reported in Fig S7B and all the TC scores are also available online (see data availability statement). For unknown reasons, two molecules escaped the previous filtering and were found to be identical, meaning they were present in both libraries (Fig S7A). One is an antifungal called clioquinol. The other compound, cetylpyridinium, is an antimicrobial used in oral health products to promote gingival health. The screen also identified several compounds in the subset of the FDA library that were similar in structure to compounds in the training set (Fig S7B); however, none of the compounds was identified as active.

Minor Comments:

(1) The compound structures in Fig 3 and Fig 6 are not clear enough.

We appreciate the reviewer's feedback. Figures 3 and 6 have now been modified to make the structures of the compounds clearer.

(2) Is the training dataset in this study available?

The data that was deemed relevant at the time of publishing the original HTS screen is available at https://doi.org/10.1371/journal.pone.0128587
However, the raw data, which was used as the training set for the current manuscript was not made available at that time. We have made it available through GitHub. Please, see Code and Data Availability" section at the end of the manuscript (see page 32, line 569-573)
